# Vitamin D Level in Patients with Consecutive Acute Coronary Syndrome Is Not Correlated with the Parameters of Platelet Activity

**DOI:** 10.3390/jcm11030707

**Published:** 2022-01-28

**Authors:** Ewelina A. Dziedzic, Jakub S. Gąsior, Izabela Sowińska, Marek Dąbrowski, Piotr Jankowski

**Affiliations:** 1 Medical Faculty, Lazarski University in Warsaw, 02-662 Warsaw, Poland; 2 Department of Internal Medicine and Geriatric Cardiology, Centre of Postgraduate Medical Education, 01-813 Warsaw, Poland; piotrjankowski@interia.pl; 3 Department of Pediatric Cardiology and General Pediatrics, Medical University of Warsaw, 02-091 Warsaw, Poland; jgasior@wum.edu.pl; 4 Medical Faculty, Medical University of Warsaw, 02-091 Warsaw, Poland; sowinska.izabela@gmail.com; 5 Department of Cardiology, Bielanski Hospital, 01-809 Warsaw, Poland; mardab@vp.pl; 6 Institute of Cardiology, Jagiellonian University Medical College, 31-008 Krakow, Poland

**Keywords:** myocardial infarction, acute coronary syndrome, vitamin D, 25-hydroxyvitamin D, platelets, atherothrombosis

## Abstract

Coronary artery disease continues to be the leading cause of death in developed countries. Elevated mean platelet volume (MPV) is associated with an increased incidence of myocardial infarction (MI) and MI-related mortality. Vitamin D concentrations affect the level and function of platelets, which are the crucial mediator of atherothrombosis and plaque rupture. The main aim of this study was to examine the relationship of serum 25-hydroxyvitamin D (25(OH)D) levels with the platelet activity in patients with a history of an acute coronary syndrome (ACS). This prospective study recruited 268 patients with a history of MI who underwent coronary angiography due to the suspicion of another ACS. Serum 25(OH)D concentration was determined by electrochemiluminescence. Platelet activity was assessed using the MPV and platelet-large cell ratio (P-LCR) parameters. There was no significant difference in MPV and P-LCR values between patients diagnosed with subsequent MI and patients with chronic coronary syndrome (CCS). A significantly lower level of 25(OH)D was demonstrated in patients who had another MI compared to those with CCS (*p* < 0.05). No significant correlation of 25(OH)D concentrations with platelet activity parameters values was found. The subgroup of patients with consecutive MI was characterized by significantly lower serum vitamin D levels, but this was not related to the analyzed parameters of platelet activity.

## 1. Introduction

Platelets are the key factor that links inflammation with thrombosis, thus being an important part of the atherosclerotic process [1]. The measurement of the thrombocyte size (mean platelet volume, MPV) and the percentage of platelet count (platelet-large cell ratio, P-LCR) are employed to assess the degree of thrombocyte stimulation, which is reflected in the process of blood clotting. MPV corresponds with the average platelet size and normally ranges from 7.5 to 10.5 fL, while P-LCR is the percentage of platelet count above 12 fL [2,3]. 

Larger thrombocytes contain more intracellular granules, which results in a greater thrombogenic potential and, thus, in higher activity [4,5]. Certain cytokines exert a significant influence on the size and total count of circulating platelets. Interleukins 3 and 6 promote the production of larger, more potent thrombocytes [6,7]. The mean volume appears to influence the capability of platelets in atherothrombosis. 

Previous studies proved that a higher MPV increases the risk of coronary artery disease (CAD) development [8]. Higher MPV and P-LCR values were described in patients diagnosed with CAD compared to healthy subjects [8]. Moreover, increased platelet activity in chronic coronary syndrome (CCS) has not only been associated with the severity of atherosclerotic lesions [9,10] but also as one of the causes of acute coronary syndrome (ACS) [11,12]. Excessive thrombocyte stimulation may lead to severe thrombotic complications including unstable angina (UA) and myocardial infarction (MI) as well as sudden cardiac death [13].

At present, MPV is considered to be an independent factor related to the lack of myocardial reperfusion, despite the restoration of the coronary blood flow [14,15,16,17]. An association between MPV and non-reflow phenomenon was established with a cut-off point of 9.05 fL [14,15,16,17]. Studies have shown an independent relationship between MPV and myocardial reperfusion disorders in nearly 40% of patients with ACS treated with percutaneous coronary intervention. It was also proven to increase both in-hospital and long-term mortality [18]. The available evidence also suggests that elevated MPV is associated with other disease states such as hypertension [19], atrial fibrillation [20], diabetes [21], chronic kidney disease [22], and obesity [23].

Vitamin D modifies the immune response by stimulating the immune cells and promoting the production of cytokines [24]. The vitamin D receptor (VDR) occurs on the surface of most of the cells involved in the formation of atherosclerotic plaques and is responsible for the severity of the course of the disease [24]. It is present both on the cells of the cardiovascular system (endothelial cells, vascular smooth muscle, cardiomyocytes) [25] and cells of the immune system [26]. Calcitriol dose-dependently inhibits the production of pro-inflammatory cytokines that have a strong thrombocyte-stimulating potential [27,28]. It also promotes the activity of Tregs, Th2 lymphocytes, and anti-inflammatory cytokines while reducing the activity of Th1 lymphocytes [29,30]. Apart from the immunomodulatory effect, it was shown that the active form of vitamin D reduces the expression of the platelet adhesion molecule CD62P [31]. This discovery may have therapeutic effects in the prevention of thrombosis [31].

To date, there has been little published on the relationship between vitamin D levels and MPV [32]. The main objective of this study was to assess whether elevated MPV and P-LCR and, thus, the increased activity of thrombocytes is correlated with vitamin D levels in a cohort of patients with a history of MI.

## 2. Materials and Methods

This study is a part of a research project focused on the association between the level of vitamin D and the severity of coronary artery atherosclerosis in Polish cardiac patients. Recently, we showed pooled data of Polish patients hospitalized in the Cardiology Department who underwent diagnostic catheter angiography for the evaluation of coronary artery disease in the years 2013–2017 [33].

The results of analyses are presented in previously published articles where details of the study population and measurements (ACS and/or diabetes diagnosis, interview questionnaire, body mass index (BMI), concentration of total cholesterol (TC) and/or triglycerides (TGs), systolic and diastolic blood pressure, coronary angiography, and total 25(OH)D in participant serum and plasma) are described [34,35,36,37].

### 2.1. Population

In this study, the results of 268 patients aged 36–93 years with a history of a previous myocardial infarction treated with acetylsalicylic acid (181 men and 87 women) were included in the final statistical analysis. The studied group of patients was treated with comparable doses of statins (i.e., atorva or rosuvastatin).

Exclusion criteria were a platelet count < 100 or >450 × 10^3^ μL; calcium–phosphate metabolism disorders, kidney disease (stage III and higher); active neoplastic process or paraneoplastic syndrome; increased values of inflammatory markers defined as a concentration of C-reactive protein > 5 mg/L and/or a total white blood cell count > 10,000 cells/μL; taking medications or dietary supplements with vitamin D and/or calcium.

### 2.2. Examinations

The biochemical tests of fasting blood from the cephalic vein were performed in a hospital laboratory using standard clinical–chemical tests. The serum concentration of 25(OH)D was determined with a DiaSorin LIAISON^®^ 25 OH Vitamin D TOTAL Assay (DiaSorin, Stillwater, MN, USA) using a chemiluminescent immunoassay (CLIA) (range of detection: 4–150 ng/mL; precision: 5.0% CV; accuracy standard deviation: 1.2% [38]). The sensitivity and the coefficient of variation (CV) of this assay was 4.01 ng/mL and 18.5%, respectively. It was shown that the within-run CV of the Elecsys Vitamin D Total assay was ≤7%, the within-laboratory CV < 9.5%, the between-laboratory precision CV ≤ 10.1%, and functional sensitivity < 9.8 nmol/L [39].

The Elecsys Vitamin D Total Assay is comparable to liquid chromatography tandem mass spectrometry and appropriate for clinical use [40,41,42]. The 25(OH)D concentrations were measured in ng/mL (1 ng/mL is equivalent to 2.5 nmol/L [43,44]).

Vitamin D status was classified according to the Endocrine Society’s clinical practice guidelines for vitamin D deficiency: a concentration of 25(OH)D < 10 ng/mL was considered as a severe deficiency, ≥10 to <20 ng/mL as a moderate deficiency, ≥20 to <30 ng/mL as a mild deficiency, and ≥30 ng/mL as optimal [45]. Participants in our study were examined throughout the whole year. Examination data corresponded to the season of blood draw: winter months (November to April) and summer months (May to October) [46]. The Republic of Poland is a country located in Central Europe with a population of approximately 38.5 million people. Poland’s capital and largest metropolis is Warsaw (52°13′ N, 21°02′ E). Vitamin D can only be produced when there is UVB, which is present for only six months in Poland [47].

A coronary angiography was performed via radial or femoral artery access. The examination with the use of contrast and X-rays allowed for the assessment of the stenosis in the coronary arteries. The severity of coronary atherosclerosis was assessed by three independent cardiologists (visual assessment), and the CASS (Coronary Artery Surgery Study Score) was used for the classification [48]. In diagnostically difficult cases (moderate/significant stenosis of the coronary artery), fractional flow reserve (FFR) was used. CASSS is a four-point (0–3) scale, the final score being the sum of points reflects one-, two-, or three-vessel CAD [48]. A score of 1 was given for >70% stenosis of one of the major coronary arteries (right coronary artery, circumflex branch, or anterior descending branch). A minimum of 50% stenosis of the left main coronary artery was scored as two points. Acute coronary syndrome was diagnosed on the basis of the guidelines of the European Society of Cardiology [49]. The main criteria for the diagnosis of ACS were the increased concentration of markers of myocardial injury and the coexistence of at least one of the criteria mentioned (i.e., symptoms of stenocardia, ECG changes suggestive of ischemia, results of imaging tests showing myocardial necrosis, or coronary artery thrombus identified on coronary angiography).

The MPV is a precise measurement of platelet size and was calculated by hematology analyzers from the volume distribution during a routine blood count. The percentage of platelets with a size of more than 12.0 fL was defined as P-LCR.

### 2.3. Statistical Analysis

The Shapiro–Wilk test was used to evaluate the data distribution. To compare the results of continuous variables between the two groups, a Mann–Whitney Test or a *t*-test were used. Pearson’s chi-squared test or Fisher’s exact test were used to determine differences between prevalence in selected groups. Logistic regression analysis was used to assess the relationship between the cause of hospitalization and selected variables. The Spearman’s correlation coefficient (*R*) was calculated to illustrate the relationship among selected variables. A two-sided *p*-value < 0.05 was considered statistically significant. The statistical analysis was performed with STATISTICA 13 (StatSoft Inc., Tulsa, OK, USA). Figures were created using GraphPad Prism 8.0 (GraphPad Software, San Diego, CA, USA).

## 3. Results

### 3.1. Participant Characteristics 

**Figure 1:**
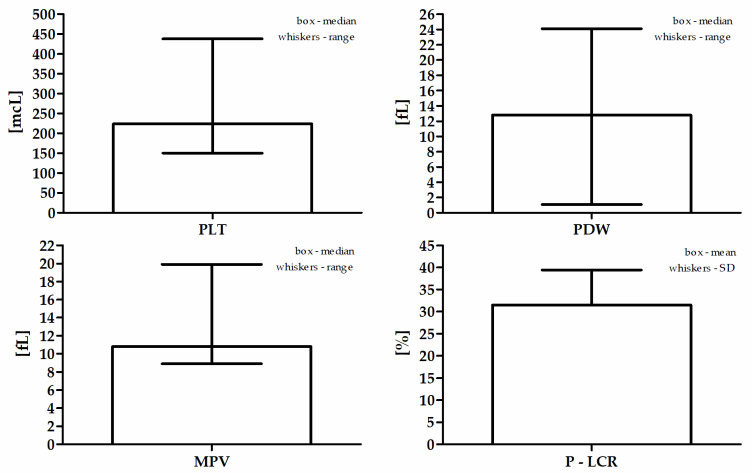
Participants’ detailed blood count data.

The mean age of the study population was 67.0 (±11.2) years. The mean BMI value was assessed among 247 patients and was 28.0 (±4.4) kg/m^2^. Sixty-one (23%) participants had a normal body weight, 111 (41%) were overweight, and 75 (28%) patients were classified as obese. A history of diabetes or diagnosis during the then current hospitalization was found in 100 (37%) patients and pre-diabetes in 10 (4%) patients. On the basis of the lipid profile, hyperlipidemia was diagnosed in over half of the patients, despite statin treatment, i.e., in 145 (54%). Hypertension was present in 233 (87%) patients. Acute coronary syndrome as the cause of hospitalization was diagnosed in 160 (60%) patients (including NSTEMI in 63–24%, STEMI in 70–26%, and UA in 27–10%), while stable CAD was the cause in 108 (40%) patients. Active smoking during the study was declared by 85 (32%) patients, and 28 (11%) patients had smoked in the past. The study was carried out in the period from October to April in a group of 200 (75%) patients, and in the months from May to September in a group of 68 (25%) patients. Insignificant changes in the coronary arteries (CASSS 0) were found only in 13 (5%) patients. One-vessel coronary disease (CASSS 1) was found in 79 (30%) patients, two-vessel (CASSS 2) in 83 (31%), and three-vessel (CASSS 3) in 93 (35%) patients. The median serum 25(OH)D level in the entire study group was 14.0 ng/mL (4.0–48.3 ng/mL). The optimal level of 25(OH)D was found in only 14 (5%) subjects. A slight deficiency was noted in 50 (19%) patients, moderate in 134 (50%) patients, while 70 (26%) respondents were qualified to the severe vitamin D deficiency group. Detailed blood count data are presented in Table 1 and Figure 1.

### 3.2. Comparison between Patients with Stable Coronary Artery Disease and Patients with Acute Coronary Syndrome

Table 2 presents the factors influencing the cause of hospitalization.

Age, sex, smoking, and diabetes were the significant factors influencing the cause of hospitalization in the described group of patients. Table 3 presents the results for selected clinical parameters in the group of patients with stable CAD and patients with ACS. 

There were statistically significant differences between patients with stable CAD and patients with ACS in the following parameters: vitamin D level and LDL level. There were statistically significant disproportions between the subgroups of patients in terms of sex, diabetes, hyperlipidemia, and smoking. Table 4 presents the results of the obtained results for selected blood count parameters in the group of patients with stable CAD and patients with ACS.

### 3.3. Correlation between Vitamin D Levels and Platelet Activity

**Figure 2:**
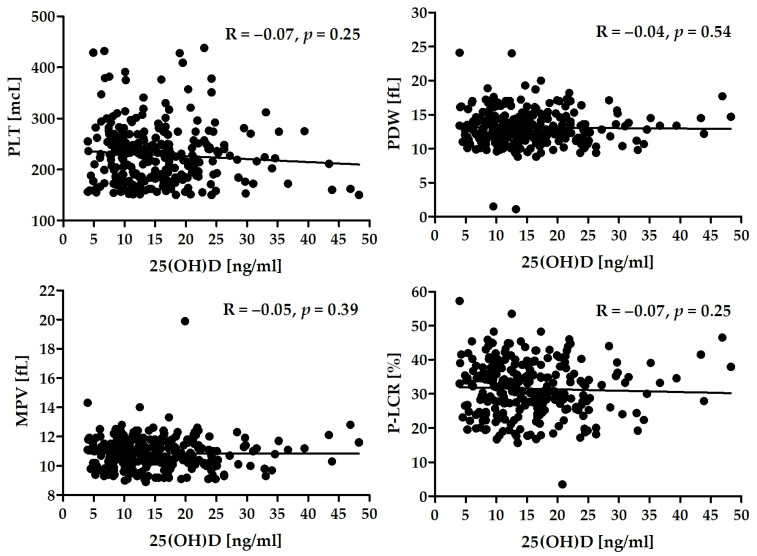
Correlation between serum 25(OH)D and selected platelet activity parameters.

There was a lack of significant correlation between the level of 25(OH)D and platelet activity in the whole group (Figure 2) as well as in the subgroups of patients (Table 5).

## 4. Discussion

In this study, we examined MPV and P-LCR values and serum 25(OH)D levels in a group of patients with a previous medical history of MI. Our current experiment substantiates previous findings in the literature. We confirmed that serum 25(OH)D concentration was significantly lower in subjects hospitalized due to the fact of subsequent MI compared with patients who had been diagnosed with CCS. Interestingly, in the studied groups of patients (subsequent ACS vs. CCS), there were no substantial differences in platelet activity parameters values. Moreover, none of the differences in the MPV and P-LCR values between patients hospitalized due to the fact of STEMI, NSTEMI, and UA were found to be statistically significant. In the analyzed group, over 95% of patients had one-, two-, or three-vessel CAD, and subsequent MI was the cause of hospitalization in nearly 60% of patients.

Numerous studies have demonstrated a direct proportion between the MPV value and the exacerbation of CAD symptoms. Patients with a diagnosis of CAD presented with an elevated mean thrombocyte size compared to the healthy population [8]. Analogously, patients diagnosed with ACS had higher MPV values than those with CCS [11,12]. Moreover, several authors have proven that elevated MPV was associated with a poor prognosis after MI [14,15,16,17,18]. A recent large meta-analysis determined the differences in mean thrombocyte size in groups of patients with an acute coronary event and diagnosis of CCS (0.84 and 0.46 fL, respectively). Patients with an MPV ≥ 7.3 fL were identified to have a two-fold higher chance of developing CAD [50]. Chu et al. revealed significant elevated MPV values in patients with MI compared with stable CAD (*p* < 0.001), and a healthy control group (*p* < 0.001). However, in comparison to patients with UA, no substantial difference in MPV value was found (*p* = 0.24) [51]. Perhaps the lack of a significant difference in the platelet activity parameters assessed in the present study results from the nature of the analyzed group. The study included patients after myocardial infarction, where in nearly 95% of patients, a significant stenosis was found in at least one of the main coronary arteries.

Few studies have been conducted so far to assess the relationship between MPV and 25(OH)D concentration. Cumhur Cure et al. have shown a statistically significant inverse relationship between MPV values and 25(OH)D concentrations in healthy subjects (*p* < 0.001) [32]. This finding was also confirmed in a group of patients with stable coronary artery disease [52] and in women with primary ovarian failure (*p* < 0.001) [53]. The obtained results indicate the impact vitamin D may have on the thrombogenic potential of platelets, thus being an individual risk factor of cardiovascular events. However, our results do not seem to confirm their observation [32,53]. In fact, they indicate a lack of influence of vitamin D on the activity of platelets. A study by Verdoia et al. has also presented the lack of 25(OH)D association with thrombocyte activity in patients treated with ASA in which the incidence of high platelet reactivity was low and independent of the 25(OH)D concentration. The opposite relationship was found in the case of P2Y12 receptor inhibitors (i.e., clopidogrel or ticagrelor) [54]. Significantly lower vitamin D levels in the MI subgroup demonstrated in the research are confirmed in cohort studies [55,56,57]. The concentration of 25(OH)D < 15 ng/mL was found to be associated with an almost two-fold higher risk of ACS. Ng et al. suggested that low vitamin D levels and a history of myocardial infarction increase the risk of recurrent major adverse cardiovascular events (MACE) including re-occurrence of ACS [56]. On the other hand, a 25(OH)D level above 7.3 ng/mL reduced the risk of non-fatal MACE in nearly 40% of patients with MI [56]. 

We are aware that our study has several limitations. Indirect, easily accessible platelet activity (i.e., MPV and P-LCR) assessment methods were used. More specific, high-priced methods of assessment could change the obtained results. In addition, thrombocytes are one of the many elements involved in the process of blood clotting on a ruptured atherosclerotic plaque, leading to the formation of a local thrombus underlying a heart attack. Perhaps more detailed and expensive methods, such as viscoelastic tests (VEAs), would change the results [58]. These methods, including thromboelastography (TEG) and rotational thromboelastometry (ROTEM), allow for the quantitative and qualitative measurement of the function of almost all components of clot formation and lysis including platelets, other blood cell components, fibrinogen, and clotting factors. The relationship between low vitamin D concentrations and reduced drug efficacy or resistance, as discussed in the medical world, may also have an impact on the presented results. Studies reported insufficient inhibition of thrombocyte aggregation by clopidogrel and ticagrelor in patients with lower vitamin D levels [54] or even clopidogrel resistance [59]. A significant association has also been demonstrated between severe vitamin D deficiency and higher platelet reactivity in diabetic patients receiving dual antiplatelet therapy (aspirin and clopidogrel or ticagrelor or prasugrel) [60]. Therefore, in our opinion, the use of VEA in the analyzed group of patients (group treated with ASA) could be even more justified. The phenomenon of platelet resistance to ASA was not considered. Another limitation of the present study was fact that the phenomenon of ASA resistance of platelets was not taken into account. The relationship between vitamin D levels and platelet activity parameters is nonlinear, which make analyses more complicated. Comorbidities other than CAD and factors that significantly affect the MPV value (e.g., diabetes, pre-diabetes, hypertension, obesity, metabolic syndrome, smoking, and lipid-lowering therapy) were not taken into account. The study included a relatively small number of patients, residents of only central Poland, mostly inhabitants of urban areas. The classification of the severity of atherosclerosis was based on coronary angiography, which does not take into account the occurrence of calcifications of the arteries that stabilize atherosclerotic plaques. All study participants were treated with a statin, but the study did not take into account the dose and duration of treatment. The presented study was cross-sectional and observational; consequently, it demonstrated a statistical association but cannot prove causation. 

In addition to the well-documented effects of vitamin D on skeletal health, low 25(OH)D levels appear to increase the risk of cardiovascular disease. The causal relationship between this group of steroid hormones and cardiovascular mortality is still under discussion. The previous randomized controlled clinical trials have not demonstrated a beneficial effect of supplementation with this substance [61]; however, the studies most often included people without clinical signs of vitamin D deficiency [62,63]. On the other hand, the presented results of nonlinear MR analysis conducted in UK Biobank (44,519 patients with CVD and 251,269 in the control group) showed an L-shaped relationship of genetically predicted serum 25(OH)D concentration with the risk of CVD and blood pressure [64]. These results suggest that improving vitamin D status in patients with low concentrations may reduce the risk of CVD. Therefore, we propose considering vitamin D deficiency as an easily modifiable risk factor for CAD in patients after myocardial infarction. 

## 5. Conclusions

In the group of patients with a history of MI treated with ASA, there were no significant differences in the parameters of platelet activity (i.e., MPV and P-LCR) regardless of the reason for hospitalization (subsequent MI vs. stable CAD). We found no significant correlation between serum 25(OH)D concentration and MPV or P-LCR. However, in patients with a consecutive coronary event, significantly lower serum concentrations of 25(OH)D were observed. This finding suggests the impact that this compound may have, despite ASA therapy, in the development of subsequent coronary events and requires detailed investigation. 

## Figures and Tables

**Table 1:** Participants’ detailed blood count data.

Parameter	Values
PLT (mcL) ^a^	224 (150–438)
PDW (fL) ^a^	12.8 (1.1–24.1)
MPV (fL) ^a^	10.8 (8.9–19.9)
P-LCR (%) ^b^	31.5 ± 8.0

PLT—platelet count (10^9^/L); PDW—platelet distribution width; MPV—mean platelet volume; P-LCR—platelet-large cell ratio. ^a^ Median and range; ^b^ mean and SD.

**Table 2:** Factors influencing the cause of hospitalization.

	Factor	Estimate	Wald Statistics	95% CI	*p*-Value
Cause of hospitalization	Sex	0.42	5.74	0.08–0.76	<0.05
	Age	−0.04	5.04	−0.07–−0.004	<0.05
	BMI	0.01	0.14	−0.06–0.09	0.71
	Diabetes mellitus	6.26	383.3	5.63–6.88	<0.001
	Hyperlipidemia	−0.02	0.01	−0.34–0.30	0.91
	Hypertension	−0.28	1.63	−0.72–0.15	0.20
	Smoking	0.88	9.92	0.33–1.42	<0.01
	CASSS	−0.06	0.04	−0.62–0.50	0.84
	Serum 25(OH)D	−0.01	0.09	−0.05–0.03	0.76
	Season during the examination	0.05	0.06	−0.31–0.41	0.80
	MPV	−0.12	0.10	−0.88–0.63	0.75
	P-LCR	−0.02	0.09	−0.11–0.08	0.76

95% CI—95% confidence interval; BMI—body mass index; CASSS—Coronary Artery Surgery Study Score.

**Table 3:** Comparison of the obtained parameters between patients with stable coronary artery disease and patients with acute coronary syndrome.

Variable	Stable CAD	ACS	*p*-Value
N	108	160	-
Sex (♀/♂)	27/81	60/100	<0.05
Age (years)	68.4 ± 9.4	66.1 ± 12.2	0.10
BMI (kg/m^2^)	27.7 ± 4.3	28.3 ± 4.6	0.34
BMI class (1/2/3) *	28/52/28	33/59/47	0.41
Diabetes (no/yes/pre-diabetes)	63/36/9	95/64/1	<0.01
TC (mg/dL)	162.5 (84.8–327.3)	171.9 (70.9–338.3)	0.07
HDL (mg/dL)	46.5 (14.6–113.2)	44.5 (19.5–92.9)	0.11
LDL (mg/dL)	81.9 (27.3–257.9)	101.4 (24.4–244.3)	<0.05
TG (mg/dL)	111.8 (37.9–417.0)	115.4 (42.6–391.8)	0.58
Hyperlipidemia (no/yes)	54/50	53/95	<0.05
Hypertension (no/yes)	15/93	20/140	0.74
Smoking (no/yes/ex-smokers)	62/24/22	93/61/6	<0.001
CASSS (0/1/2/3)	6/25/39/38	7/54/44/55	0.24
Serum 25(OH)D (ng/mL)	15.8 (4.0–46.9)	13.1 (4.0–48.3)	<0.05
Season of the examination(November to April/May to October)	78/30	122/38	0.46

* 1—<25; 2—25–30; 3—>30.

**Table 4:** Differences in platelet activity parameters between patients with stable CAD and MI.

Parameter	Stable CAD	MI	*p*-Value
PLT (mcL)	216 (150–438)	227 (150–432)	0.20
PDW (fL)	12.9 (8.8–24.1)	12.8 (1.1–20.0)	0.24
MPV (fL)	10.9 (9.1–19.9)	10.8 (8.9–13.3)	0.32
P-LCR (%)	32.1 ± 8.3	31.0 ± 7.7	0.26

PLT—platelet count (10^9^/L); PDW—platelet distribution width; MPV—mean platelet volume; P-LCR—platelet-large cell ratio.

**Table 5:** Correlation between serum 25(OH)D and selected parameters in both groups of patients.

	Stable CAD	ACS
PLT (mcL)	*R* = −0.07, *p* = 0.49	R = −0.05, *p* = 0.53
PDW (fL)	*R* = −0.11, *p* = 0.25	*R* = −0.01, *p* = 0.89
MPV (fL)	R = −0.13, *p* = 0.19	*R* = −0.01, *p* = 0.86
P-LCR (%)	*R* = −0.13, *p* = 0.18	*R* = −0.04, *p* = 0.61

PLT—platelet count (10^9^/L); PDW—platelet distribution width; MPV—mean platelet volume; P-LCR—platelet-large cell ratio.

## Data Availability

Data can be provided by the corresponding author upon reasonable request.

## References

[1] Gawaz M., Langer H., May A.E. (2005). Platelets in inflammation and atherogenesis. J. Clin. Investig..

[2] Park Y., Schoene N., Harris W. (2002). Mean platelet volume as an indicator of platelet activation: Methodological issues. Platelets.

[3] Varol E., Ozaydin M., Türker Y., Alaca S. (2009). Mean platelet volume, an indicator of platelet activation, is increased in patients with mitral stenosis and sinus rhythm. Scand. J. Clin. Lab. Investig..

[4] Desai K.N., Patel K., Shah M., Ranapurwala M., Chaudhari S., Shah M., Shah M. (2013). A study of platelet volume indices (PVI) in patients of coronary artery disease and acute myocardial infarction in tertiary care hospital. Int. J. Adv. Res..

[5] Kanbay A., Tutar N., Kaya E., Buyukoglan H., Ozdogan N., Oymak F.S., Gulmez I., Demir R. (2013). Mean platelet volume in patients with obstructive sleep apnea syndrome and its relationship with cardiovascular diseases. Blood Coagul. Fibrinolysis.

[6] Debili N., Massé J.M., Katz A., Guichard J., Breton-Gorius J., Vainchenker W. (1993). Effects of the recombinant hematopoietic growth factors interleukin-3. interleukin-6. stem cell factor. and leukemia inhibitory factor on the megakaryocytic differentiation of CD34+ cells. Blood.

[7] Burstein S.A., Downs T., Friese P., Lynam S., Anderson S., Henthorn J., Epstein R.B., Savage K. (1992). Thrombocytopoiesis in normal and sublethally irradiated dogs: Response to human interleukin-6. Blood.

[8] Khandekar M.M., Khurana A.S., Deshmukh S.D., Kakrani A.L., Katdare A.D., Inamdar A.K. (2006). Platelet volume indices in patients with coronary artery disease and acute myocardial infarction: An Indian scenario. J. Clin. Pathol..

[9] Murat S.N., Duran M., Kalay N., Gunebakmaz O., Akpek M., Doger C., Elcik D., Ocak A., Vatankulu M.A., Turfan M. (2013). Relation between mean platelet volume and severity of atherosclerosis in patients with acute coronary syndromes. Angiology.

[10] Ekici B., Erkan A.F., Alhan A., Sayın I., Aylı M., Töre H.F. (2013). Is mean platelet volume associated with the angiographic severity of coronary artery disease?. Kardiol. Pol..

[11] Pal R., Bagarhatta R., Gulati S., Rathore M., Sharma N. (2014). Mean platelet volume in patients with acute coronary syndromes: A supportive diag-nostic predictor. J. Clin. Diagn. Res..

[12] Khode V., Sindhur J., Kanbur D., Ruikar K., Nallulwar S. (2012). Mean platelet volume and other platelet volume indices in patients with stable coronary artery disease and acute myocardial infarction: A case control study. J. Cardiovasc. Dis. Res..

[13] Gawlita M., Wasilewski J., Osadnik T., Reguła R., Bujak K., Gonera M. (2015). Mean platelet volume and platelet-large cell ratio as prognostic factors for coronary artery disease and myocardial infarction. Folia Cardiol..

[14] Rechciński T., Jasińska A., Foryś J., Krzemińska-Pakuła M., Wierzbowska-Drabik K., Plewka M., Peruga J.Z., Kasprzak J.D. (2013). Prognostic value of platelet indices after acute myocardial infarction treated with primary percutaneous coronary intervention. Cardiol. J..

[15] Kırbaş Ö., Kurmuş Ö., Köseoğlu C., Duran Karaduman B., Saatçi Yaşar A., Alemdar R., Ali S., Bilge M. (2014). Association between admission mean platelet volume and ST segment resolution after thrombolytic therapy for acute myocardial infarction. Anadolu. Kardiyol. Derg..

[16] Sarli B., Baktir A.O., Saglam H., Arinc H., Kurtul S., Sivgin S., Akpek M., Kaya M.G. (2013). Mean platelet volume is associated with poor postinterventional myocardial blush grade in patients with ST-segment elevation myocardial infarction. Coron. Artery Dis..

[17] Sezer M., Okcular I., Goren T., Oflaz H., Nisanci Y., Umman B., Mercanoglu F., Bilge A.K., Meric M., Umman S. (2007). Association of haematological indices with the degree of microvascular injury in patients with acute anterior wall myocardial infarction treated with primary percutaneous coronary intervention. Heart.

[18] Slavka G., Perkmann T., Haslacher H., Greisenegger S., Marsik C., Wagner O.F., Endler G. (2011). Mean platelet volume may represent a predictive parameter for overall vascular mortality and ischemic heart disease. Arter. Thromb. Vasc. Biol..

[19] Gang L., Yanyan Z., Zhongwei Z., Juan D. (2017). Association between mean platelet volume and hypertension incidence. Hypertens. Res..

[20] Tekin G., Tekin Y.K., Sivri N., Yetkin E. (2013). Mean platelet volume in patients with nonvalvular atrial fibrillation. Blood Coagul. Fibrinolysis.

[21] Hekimsoy Z., Payzin B., Ornek T., Kandoğan G. (2004). Mean platelet volume in Type 2 diabetic patients. J. Diabetes Complicat..

[22] Ju H.Y., Kim J.K., Hur S.M., Woo S.A., Park K.A., Park M.Y., Choi S.J., Hwang S.D. (2015). Could mean platelet volume be a promising biomarker of progression of chronic kidney disease?. Platelets.

[23] Coban E., Ozdogan M., Yazicioglu G., Akcit F. (2005). The mean platelet volume in patients with obesity. Int. J. Clin. Pract..

[24] Kassi E., Adamopoulos C., Basdra E., Papavassiliou A.G. (2013). Role of vitamin D in atherosclerosis. Circulation.

[25] Brandenburg V.M., Vervloet M.G., Marx N. (2012). The role of vitamin D in cardiovascular disease: From present evidence to future perspectives. Atherosclerosis.

[26] Prietl B., Treiber G., Pieber T.R., Amrein K. (2013). Vitamin D and immune function. Nutrients.

[27] Müller K., Haahr P.M., Diamant M., Rieneck K., Kharazmi A., Bendtzen K. (1992). 1.25-Dihydroxyvitamin D3 inhibits cytokine production by human blood monocytes at the post-transcriptional level. Cytokine.

[28] Talmor Y., Golan E., Benchetrit S., Bernheim J., Klein O., Green J., Rashid G. (2008). Calcitriol blunts the deleterious impact of advanced glycation end products on en-dothelial cells. Am. J. Physiol. Renal Physiol..

[29] Young J.L., Libby P., Schönbeck U. (2002). Cytokines in the pathogenesis of atherosclerosis. Thromb. Haemost..

[30] Robertson A.K., Hansson G.K. (2006). T cells in atherogenesis: For better or for worse?. Arterioscler. Thromb. Vasc. Biol..

[31] Stach K., Kälsch A.I., Nguyen X.D., Elmas E., Kralev S., Lang S., Weiss C., Borggrefe M., Kälsch T. (2011). 1α,25-Dihydroxyvitamin D3 attenuates platelet activation and the expression of VCAM-1 and MT1-MMP in human endothelial cells. Cardiology.

[32] Cumhur Cure M., Cure E., Yuce S., Yazici T., Karakoyun I., Efe H. (2014). Mean platelet volume and vitamin D level. Ann. Lab. Med..

[33] Dziedzic E.A., Gąsior J.S., Saniewski T. (2021). Dąbrowski, M. Vitamin D deficiency among Polish patients with angiographically confirmed coronary heart disease. Pol. Merkur. Lek..

[34] Dziedzic E.A., Gąsior J.S., Pawłowski M., Dąbrowski M. (2017). Association of Vitamin D Deficiency and Degree of Coronary Artery Disease in Cardiac Patients with Type 2 Diabetes. J. Diabetes Res..

[35] Dziedzic E.A., Gąsior J.S., Pawłowski M., Wodejko-Kucharska B., Saniewski T., Marcisz A., Dąbrowski M.J. (2019). Vitamin D level is associated with severity of coronary artery atherosclerosis and incidence of acute coronary syndromes in non-diabetic cardiac patients. Arch. Med. Sci..

[36] Dziedzic E.A., Przychodzeń S., Dąbrowski M. (2016). The effects of vitamin D on severity of coronary artery atherosclerosis and lipid profile of cardiac patients. Arch. Med. Sci..

[37] Dziedzic E.A., Smyk W., Sowińska I., Dąbrowski M., Jankowski P. (2021). Serum Level of Vitamin D Is Associated with Severity of Coronary Atherosclerosis in Postmenopausal Women. Biology.

[38] Krzywanski J., Mikulski T., Krysztofiak H., Mlynczak M., Gaczynska E., Ziemba A. (2016). Seasonal vitamin D status in polish elite athletes in relation to sun exposure and oral supplementation. PLoS ONE.

[39] Wielders J.P., Carter G.F., Eberl H., Morris G., Roth H.J., Vogl C. (2015). Automated Competitive Protein-Binding Assay for Total 25-OH Vitamin D. Multicenter Evaluation and Practical Performance. J. Clin. Lab. Anal..

[40] Abdel-Wareth L., Haq A., Turner A., Khan S., Salem A., Mustafa F., Hussein N., Pallinalakam F., Grundy L., Patras G. (2013). Total vitamin D assay comparison of the Roche Diagnostics “Vitamin D total” electrochemiluminescence protein binding assay with the Chromsystems HPLC method in a population with both D2 and D3 forms of vitamin D. Nutrients.

[41] Knudsen C.S., Nexo E., Højskov C.S., Heickendorff L. (2012). Analytical validation of the Roche 25-OH Vitamin D Total assay. Clin. Chem. Lab. Med..

[42] Shin S.Y., Kwon M.J., Song J., Park H., Woo H.Y. (2013). Measurement of serum total vitamin D (25-OH) using automated immunoassay in comparison [corrected] with liquid chromatography tandem-mass spectrometry. J. Clin. Lab. Anal..

[43] Sahota O. (2014). Understanding vitamin D deficiency. Age Ageing.

[44] Heaney R.P. (2008). Vitamin D in health and disease. Clin. J. Am. Soc. Nephrol..

[45] Holick M.F., Binkley N.C., Bischoff-Ferrari H.A., Gordon C.M., Hanley D.A., Heaney R.P., Murad M.H., Weaver C.M. (2011). Evaluation, treatment, and prevention of vitamin D deficiency: An Endocrine Society clinical practice guideline. J. Clin. Endocrinol. Metab..

[46] Al-Khalidi B., Kimball S.M., Rotondi M.A., Ardern C.I. (2017). Standardized serum 25-hydroxyvitamin D concentrations are inversely associated with cardiometabolic disease in U.S. adults: A cross-sectional analysis of NHANES, 2001–2010. Nutr. J..

[47] Engelsen O. (2010). The relationship between ultraviolet radiation exposure and vitamin D status. Nutrients.

[48] Ringqvist I., Fisher L.D., Mock M., Davis K.B., Wedel H., Chaitman B.R., Passamani E., Russell R.O., Alderman E.L., Kouchoukas N.T. (1983). Prognostic value of angiographic indices of coronary artery disease from the Coronary Artery Surgery Study (CASS). J. Clin. Investig..

[49] Collet J.P., Thiele H., Barbato E., Barthélémy O., Bauersachs J., Bhatt D.L., Dendale P., Dorobantu M., Edvardsen T., Folliguet T. (2021). 2020 ESC Guidelines for the management of acute coronary syndromes in patients presenting without persistent ST-segment elevation. Eur. Heart J..

[50] Sansanayudh N., Anothaisintawee T., Muntham D., McEvoy M., Attia J., Thakkinstian A. (2014). Mean platelet volume and coronary artery disease: A systematic review and meta-analysis. Int. J. Cardiol..

[51] Chu S.G., Becker R.C., Berger P.B., Bhatt D.L., Eikelboom J.W., Konkle B., Mohler E.R., Reilly M.P., Berger J.S. (2010). Mean platelet volume as a predictor of cardiovascular risk: A systematic review and meta-analysis. J. Thromb. Haemost..

[52] Korzonek-Szlacheta I., Hudzik B., Nowak J., Szkodzinski J., Nowak J., Gąsior M., Zubelewicz-Szkodzinska B. (2018). Mean platelet volume is associated with serum 25-hydroxyvitamin D concentrations in patients with stable coronary artery disease. Heart Vessel..

[53] Kebapcilar A.G., Kulaksizoglu M., Ipekci S.H., Korkmaz H., Kebapcilar L., Akyurek F., Taner C.E., Gonen M.S. (2013). Relationship between mean platelet volume and low-grade systemic coagulation with vitamin D deficiency in primary ovarian insufficiency. Arch. Gynecol. Obstet..

[54] Verdoia M., Pergolini P., Rolla R., Sartori C., Nardin M., Schaffer A., Barbieri L., Daffara V., Marino P., Bellomo G. (2016). Vitamin D levels and high-residual platelet reactivity in patients receiving dual antiplatelet therapy with clopidogrel or ticagrelor. Platelets.

[55] Giovannucci E., Liu Y., Hollis B.W., Rimm E.B. (2008). 25-hydroxyvitamin D and risk of myocardial infarction in men: A prospective study. Arch. Intern. Med..

[56] Ng L.L., Sandhu J.K., Squire I.B., Davies J.E., Jones D.J. (2013). Vitamin D and prognosis in acute myocardial infarction. Int. J. Cardiol..

[57] Wang T.J., Pencina M.J., Booth S.L., Jacques P.F., Ingelsson E., Lanier K., Benjamin E.J., D’Agostino R.B., Wolf M., Vasan R.S. (2008). Vitamin D deficiency and risk of cardiovascular disease. Circulation.

[58] Selby R. (2020). “TEG talk”: Expanding clinical roles for thromboelastography and rotational thromboelastometry. Hematol. Am. Soc. Hematol. Educ. Program.

[59] Lu B.C., Shi X.J., Liang L., Dong N., Liu Z.Z. (2019). Platelet Surface CD62p and Serum Vitamin D Levels are Associated with Clopidogrel Resistance in Chinese Patients with Ischemic Stroke. J. Stroke Cerebrovasc. Dis..

[60] Verdoia M., Pergolini P., Rolla R., Nardin M., Schaffer A., Barbieri L., Daffara V., Marino P., Bellomo G., Suryapranata H. (2017). Impact of high-dose statins on vitamin D levels and platelet function in patients with coronary artery disease. Thromb. Res..

[61] Barbarawi M., Kheiri B., Zayed Y., Barbarawi O., Dhillon H., Swaid B., Yelangi A., Sundus S., Bachuwa G., Alkotob M.L. (2019). Vitamin D Supplementation and Cardiovascular Disease Risks in More Than 83 000 Individuals in 21 Randomized Clinical Trials: A Meta-analysis. JAMA Cardiol..

[62] Quyyumi A.A., Al Mheid I. (2019). The Demise of Vitamin D for Cardiovascular Prevention. JAMA Cardiol..

[63] Scragg R. (2018). Emerging Evidence of Thresholds for Beneficial Effects from Vitamin D Supplementation. Nutrients.

[64] Zhou A., Selvanayagam J.B., Hyppönen E. (2021). Non-linear Mendelian randomization analyses support a role for vitamin D deficiency in cardiovascular disease risk. Eur. Heart J..

